# Characterizing the bacterial community across the gastrointestinal tract of goats: Composition and potential function

**DOI:** 10.1002/mbo3.820

**Published:** 2019-03-03

**Authors:** Lizhi Wang, Lei Jin, Bai Xue, Zhisheng Wang, Quanhui Peng

**Affiliations:** ^1^ Institute of Animal Nutrition Sichuan Agricultural University Chengdu PR China

**Keywords:** bacterial community, composition and function, gastrointestinal tract, goat

## Abstract

The composition and function of the microbial community in the gastrointestinal tract (GIT) have increasingly captured the attention of nutritionists because these traits affect the nutrient utilization efficiency and health of host animals. Little information has been reported on these aspects of the goat GIT. This study used 12 female goats (weighing 20.70 ± 1.60 kg and 10 months of age) to examine the composition and function of the microbiota in the rumen, abomasum, jejunum, cecum, and colon. Total genomic DNA was extracted from chyme samples from different sections of the GIT, and the hypervariable region of the 16S rRNA gene was amplified by PCR using bacterial universal primers. The amplicons were sequenced on an Illumina MiSeq platform, and the biological information was analyzed using QIIME software. A total of 857 genera that belonged to 39 phyla were observed across the goat GIT, with Bacteroidetes and Firmicutes dominating. Our results revealed significant differences in the composition, diversity, and species abundance of the bacterial communities in the different sections of the GIT. However, the compositions of the bacterial communities in adjacent GIT segments showed similarities in addition to differences. The study indicated that there were significant differences in microbial function among the GIT regions. In particular, the relative abundances of genes involved in energy metabolism, amino acid metabolism, nucleotide metabolism, and glycan metabolism were overrepresented in samples from the forestomach, and genes related to energy metabolism, amino acid metabolism, and glycan metabolism were mainly enriched in samples from the small intestine. Additionally, the relative abundances of bacteria at the phylum and genus levels were significantly correlated with these metabolic functions. In general, there were significant differences in composition and potential function among the bacterial communities in the goat GIT.

## 1 INTRODUCTION

The gastrointestinal tract (GIT) of ruminants harbors a dense and diverse microbiota that has long been recognized as an essential factor in converting plant materials into digestible substances. The existing physiological and biochemical knowledge has revealed that the bacteria in distinct regions of the GIT have different functions. For example, the microbes in the rumen mainly help the host degrade dietary components such as fiber, but the microbes in the small intestine play a significant role in maintaining the health of the host as well as in digesting nutrients (Bauer et al., 2018; Cervantesbarragan et al., 2017; Dodd et al., 2017; Kadoki et al., 2017; Koppel, Maini, & Balskus, 2017). The function of the microbiota is based on its composition and phylogenetic distribution, and the differences in composition and structure inevitably lead to the differences in function between different microbiota. Nevertheless, because of their convenience, microbiological samples derived from the rumen or feces are often used when assessing the health and digestive function of the whole GIT (Abderzak et al., 2012; Ramírez‐Restrepo et al., 2016; Riyanti, Suryahadi, & Evvyernie, 2015). Little research has been conducted to analyze the microbial composition in other GIT compartments of goats (such as in the small and large intestine) (De Oliveira et al., 2013). However, experiments in chicken (Zhao et al., 2013), donkeys (Liu et al., 2014), horses (Dougal et al., 2012), and mice (Gu et al., 2013) have shown high variation among the microbial communities of different regions of the GIT. Ruminal or fecal microbiota cannot reflect the microbial communities in other segments of the GIT (Mao, Zhang, Liu, & Zhu, 2015). The use of samples from the rumen or feces to speculate on the structure and composition of bacterial communities in other GIT compartments would not allow researchers to fully understand the microbial function of the different communities. To gain a comprehensive understanding of functional localization, the microbiota in different parts of the GIT should be analyzed.

Previous studies have found that the microbiota varied greatly with the animal species (Ley et al., 2008). Thus, although the microbial compositions in the GIT of steers (De Oliveira et al., 2013) and dairy cattle (Mao et al., 2015) have been revealed, information on the compositions, functions, and metabolic activities of the bacterial communities in the GIT of goats remains unknown (Ramírez‐Restrepo et al., 2016; Riyanti et al., 2015). In the present study, we hypothesized that the diversity and function of the microbial community in different regions along the GIT of goats varied significantly, and an experiment was conducted to characterize the compositions and distributions of the gastrointestinal microbiota in goats using high‐throughput 16S rRNA gene amplicon sequencing and to analyze their potential functional differences using PICRUSt (phylogenetic investigation of communities by reconstruction of unobserved states).

## 2 MATERIALS AND METHODS

### 2.1 Animals and sample collection

Twelve female Nubian black goats, which were 10 months old and weighed 20.70 ± 1.60 kg, were used in this study. Throughout the experimental period, the goats were fed a total mixed ration (TMR) to avoid the selection of feed components. The TMR contained 38.47% corn, 20.00% alfalfa meal, 35.00% *Leymus chinensis*, 4.50% soybean meal, 0.45% NaCl, 0.45% baking soda, 0.08% CaCo_3_, 0.60% CaHPO_4_, and 0.45% premix and had a nutritive content of 9.71% CP, 24.07% ADF, 36.11% NDF, 2.95% EE, and 9.33 MJ/kg ME on a dry matter basis. All goats were fed twice daily with equal amounts of feed at 8:00 a.m. and 5:00 p.m. and were kept in individual cages under controlled environmental conditions with free access to food and water. The experiment lasted for 60 days, including 15 days for adaptation.

On day 60, the goats were slaughtered, and the luminal contents were collected from the rumen, abomasum, jejunum, cecum, and colon (50 ml). The sampling procedure was as follows: the goats were transferred to a biopsy table postmortem. Subsequently, the rumen and abomasum were cut with sterilized scissors, and the contents of these compartments were collected. During the intestinal sampling, the jejunum, cecum, and colon were isolated by tying off each anatomical section at both ends with thread to prevent the movement of the luminal contents from one region to another. All samples were kept at −80°C until DNA extraction.

### 2.2 DNA extraction, PCR amplification, and Illumina MiSeq sequencing

Total microbial DNA was extracted from the luminal contents and purified using a method described previously (Guo et al., 2015). The quality of the DNA was determined using agarose electrophoresis and a Nanodrop 8000 spectrophotometer (Thermo Scientific, Australia). The high quality DNA was amplified using the 515F/806R primer set (forward primer 515F with a sequence of 5′‐GTGCCAGCMGCCGCGGTAA‐3′ and reverse primer 806R with a sequence of 5′‐GGACTACVSGGGTATCTAAT‐3′) (Caporaso et al., 2011) that targets the V4 hypervariable region of the bacterial 16S rRNA gene, with a unique 5‐ to 8‐base error‐correcting barcode for multiplexed DNA sequencing.

The amplification was initiated with denaturation at 94°C for 3 min, followed by 30 cycles at 94°C for 30 s, 58°C for 30 s, and 72°C for 90 s, and a last extension at 72°C for 5 min. The 50 μl reaction mixture contained 200 nM of each primer, 5 μl of 2.50 mmol/L dNTP mixture, 5 μl of 10× Ex Taq buffer (20 mmol/L Mg2^+^; Takara Inc., Dalian, China), 0.35 μg of template DNA, 2 mM of MgCl_2_, 4 units of Taq DNA polymerase (Takara Inc.), and approximately 37 μl Milli‐Q water. The amplicons were purified using a PCR Clean‐Up system (Promega, Madison) with a purification kit (QIAGEN, Australia) and were quantified using a QuantiFluor™‐ST fluorometer (Promega, China). Finally, the samples were sequenced on the MiSeq Illumina sequencing platform (Novogene Technology Co., Ltd, Beijing, China), according to the protocols described in previous article (Caporaso et al., 2012).

### 2.3 Bioinformatic analysis

Pyrosequencing reads were mainly analyzed using QIIME (version 1.8.0) pipeline software (Caporaso, Kuczynski, & Stombaugh, 2010). Sequences with an average quality of <20 over a 50 bp sliding window were removed. The UCHIME algorithm (Edgar, Haas, Clemente, Quince, & Knight, 2011) implemented in Mothur (version 1.35.1) (Schloss et al., 2009) was used to remove chimeric sequences. Sequencing noise was further reduced using a preclustering approach (Huse, Welch, Morrison, & Sogin, 2010). Uclust (version 1.2.22q) (Edgar, 2010) was then used to cluster the obtained clean and high‐quality sequences into operational taxonomic units (OTUs) for an eventual taxonomy assignment based on 97% sequence similarity (http://www.mothur.org/wiki/Greengenes-formatted_databases, gg_otu_13_8). The most abundant sequence was selected as the representative for each OTU and was assigned to a taxonomic group using RDP Classifier (version 2.12) (Cole et al., 2009).

The chimeric OTUs were removed from the analysis against the sequence from the SILVA database (Quast et al., 2013) (http://www.mothur.org/wiki/Silva-reference-files). Good's coverage and rarefaction curves were determined to estimate the coverage and sampling effort using the analysis of alpha diversity. Mothur was also used to calculate the population diversity (Simpson index), evenness (Shannon index), richness (Chao1) and phylogenetic diversity (PD).

Beta diversity was measured by calculating the weighted and unweighted UniFrac distances between each pair of samples, and the unweighted UniFrac distance matrix was measured and visualized using a principal coordinate analysis (PCoA) (Lozupone, Lladser, Knights, Stombaugh, & Knight, 2011). A PCoA was applied to the resulting distance matrices to generate two‐dimensional plots using R (version x64 3.4.2) (http://cran.rstudio.com). According to the results of the species classification, OriginPro (version 9.0) software was used to draw a relative abundance histogram of the dominant bacterial phyla. In addition, the genera that were shared by all samples were selected to create a heatmap using R (version x64 3.4.2).

Finally, the putative bacterial metabolic pathways and functions were assessed via PICRUSt (Langille et al., 2013). PICRUSt is a bioinformatics tool designed to predict the gene functions of a microbial community. The inferred genes and their functions were aligned with the Kyoto Encyclopedia of Genes and Genomes (KEGG, http://www.genome.jp/kegg/), which is a database resource for understanding the high‐level functions and utilities of biological systems. A similarity search with an *E*‐value <10^−5^ was performed for the prediction and functional annotation (Fu et al., 2016).

### 2.4 Statistical analysis

Nonparametric tests were performed using SPSS (version 20.0) for Windows (SPSS Inc., Chicago, IL) to analyze the effects of GIT region on bacterial prevalence and the relative abundance values of the KEGG pathways. The results are shown as the means ± *SD*. Correlations were determined using Spearman correlation analysis. Differences between means were considered significant at *p *<* *0.05 and extremely significantly different at *p *<* *0.01.

## 3 RESULTS

### 3.1 Data acquisition and alpha diversity analysis

We obtained 75,575 ± 4,968, 73,400 ± 5,349, 69,954 ± 6,950, 73,423 ± 4,869, and 70,584 ± 7,532 (sequences/sample) high‐quality sequences and detected 2,612 ± 233, 2,578 ± 258, 2,529 ± 471, 3,861 ± 552, and 3,347 ± 422 OTUs per sample from the chyme samples of the rumen, abomasum, jejunum, colon, and cecum, respectively, based on a 97% similarity level. The number of OTUs in the large intestine (cecum and colon) samples was far greater (*p* < 0.01) than that in the jejunum samples and in the forestomach (rumen and abomasum) samples (Table 1).

**Table 1:** Valid sequences and alpha diversity

Regions	Reads	OTUs	Chao 1	Shannon	Simpson	Good's coverage	PD
Rumen	75,575 ± 3,179	2,612 ± 77^Bb^	2,466.49 ± 157.91^Bb^	7.62 ± 0.53^Bb^	0.979 ± 0.014^Aa^	0.9941 ± 0.0008^Aa^	170.70 ± 5.71^Bc^
Abomasum	73,400 ± 3,332	2,578 ± 167^Bb^	2,497.79 ± 210.44^Bb^	7.56 ± 0.51^Bb^	0.977 ± 0.016^Aa^	0.9935 ± 0.0006^Ab^	175.24 ± 7.72^Bb^
Jejunum	69,954 ± 5,965	2,529 ± 161^Bb^	2,464.79 ± 393.92^Bb^	6.86 ± 1.25^Cc^	0.956 ± 0.038^Bb^	0.9922 ± 0.0019^Bc^	170.85 ± 4.59^Bc^
Colon	73,423 ± 3,781	3,861 ± 180^Aa^	3,733.21 ± 377.35^Aa^	8.24 ± 0.55^Aa^	0.986 ± 0.011^Aa^	0.9943 ± 0.0019^Aa^	184.74 ± 5.75^Aa^
Cecum	70,584 ± 7,084	3,347 ± 247^Aa^	3,306.76 ± 119.26^Aa^	7.94 ± 0.41^Bb^	0.988 ± 0.007^Aa^	0.9930 ± 0.0011^Ab^	184.02 ± 3.49^Aa^

Note

Values are expressed as the *M*s ± *SD*. Values within the same column with same superscripts were not significantly different from one another (*p* > 0.05); however, Values with different lowercase letter superscripts were significantly different (*p* < 0.05), and values with different capital letter superscripts were extremely significantly different (*p* < 0.01).

OTU: operational taxonomic unit; PD: phylogenetic diversity.

The alpha diversity in the large intestine samples was greater (*p* < 0.01) than that in the jejunum and forestomach samples (Table 1). The samples from the large intestine had the highest diversity, while those from small intestine had the lowest Chao 1, Shannon and Simpson values. The PD, calculated as the sum of all the branch lengths in a 16S rRNA tree, was found to be variable across the goat GIT, reaching a maximum value (*p *< 0.01) in the large intestine sample (Table 1).

**Figure 1:**
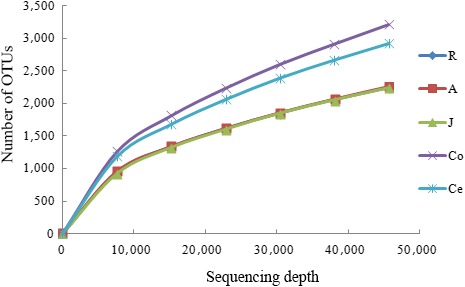
Rarefaction curve. To evaluate the sampling depth, rarefaction curves of the microbial communities based on 16S rRNA gene sequences are shown. *Note*. OTU: operational taxonomic unit; R: rumen samples; A: abomasum samples; J: jejunum samples; Co: colon samples; Ce: cecum samples

Good's coverage across the GIT was >0.99, implying that the sampling depth was sufficient to estimate the microbial diversity (Table 1). This result was confirmed by rarefaction curves (Figure 1). All the curves asymptotically approached a plateau, suggesting that the curves accurately reflected the microbial community.

### 3.2 Beta diversity analysis

**Figure 2:**
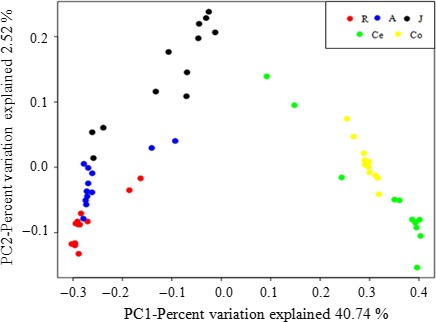
Cluster analysis by the principal coordinate analysis. The distances between the samples, which were based on similarity in operational taxonomic unit (OTU) composition (OTU similarity ≥97%) calculated using unweighted UniFrac distances, were visualized by principal coordinates analysis plots. A greater distance between two samples indicated a lower similarity. The percentage of variation explained by PC1 and PC2 are noted in the axes. *Note*. R: rumen samples; A: abomasum samples; J: jejunum samples; Co: colon samples; Ce: cecum samples

A PCoA of overall diversity based on unweighted UniFrac values was also performed to compare the microbial diversity of all samples. The analysis showed that microbial communities from the same/adjacent GIT regions (forestomach, jejunum, and large intestine) were more similar to each other than to those from other regions (Figure 2). Furthermore, the microbiota in the large intestine was clearly different from that from other regions, as shown by PC1, which accounted for 40.74% of the total variation, and the microbiota in forestomach was different from that in the jejunum, as shown by PC2, which represented 2.52% of the total variation.

### 3.3 Phylum‐ and genus‐level microbial composition

**Figure 3:**
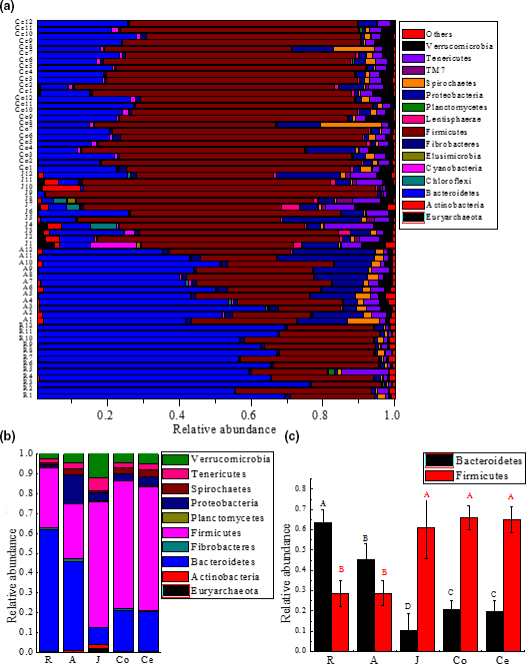
Microbial composition at the phylum level. (a) Shared phyla across the gastrointestinal tract (GIT) of goats; bar plots showing the average relative abundances of the bacterial phyla (%). (b) Depicted are the average relative abundances of the phyla (relative abundances of the top 10 phyla in at least one GIT region). (c) Comparison of the relative abundances of the two main bacterial phyla found at every sampling site, Bacteroidetes and Firmicutes, with relative abundances shown on the *Y*‐axis. *Note*. R: rumen samples; A: abomasum samples; J: jejunum samples; Co: colon samples; Ce: cecum samples

A total of 39 bacterial phyla were identified in all samples, 15 were common among the samples (Figure 3a), and Bacteroidetes and Firmicutes were the most abundant phyla in all samples (Figure 3b). The relative abundance of Bacteroidetes was the highest in the forestomach (63.62 ± 1.81% in the rumen and 45.23 ± 2.45% in the abomasum) and was significantly (*p* < 0.01) higher than that in the jejunum (10.14 ± 4.02%) and large intestine (20.46 ± 1.62% in the colon and 19.48 ± 1.56% in the cecum). The most abundant phylum in the forestomach was Bacteroidetes, while that in jejunum and large intestine was Firmicutes. The relative abundance of Firmicutes in the jejunum, colon, and cecum was 61.19 ± 5.23%, 66.05 ± 2.93%, and 64.77 ± 1.67%, respectively and was significantly higher (*p* < 0.01) than that in the forestomach (28.52 ± 1.79% in the rumen; 28.75 ± 1.71% in the abomasum, Figure 3c).

**Figure 4:**
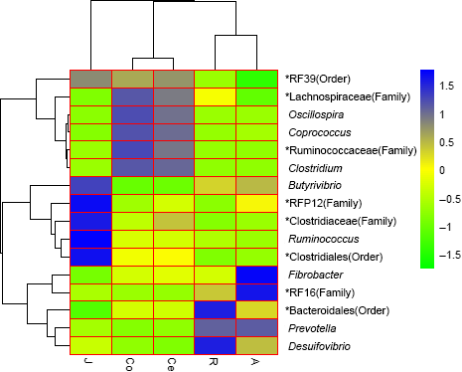
Cluster heatmap of the shared genera. *Note*. The heatmap was constructed to determine the relationship between the operational taxonomic units and experimental treatments based on log transformed relative abundances. The phylogenetic tree was constructed with maximum likelihood using FastTree 2.1.3 (*Y*‐axis clustering). Hierarchical clustering based on the distances of the five samples along the *X*‐axis and the bacterial genera along the *Y*‐axis are indicated in the upper part and on the left side of the figure, respectively. The closer to blue, the higher is the relative abundance, while the closer to green, the lower is the relative abundance. *Note*. R: rumen samples; A: abomasum samples; J: jejunum samples; Co: colon samples; Ce: cecum samples

At the genus level, a total of 857 bacterial genera were detected, and the average relative abundances of the top 10 abundant genera were compared among the GIT segments (Table 2). The proportions of *Prevotella* and *Bacteroidales (order) were higher (*p* < 0.01) in the forestomach than in the jejunum and large intestine. The proportions of *Ruminococcus*, *Clostridiales (order), and *Butyrivibrio* were higher (*p* < 0.01) in the jejunum than in the forestomach and large intestine. The proportions of *Ruminococcaceae (family), *Clostridium,* and *Lachnospiraceae (family) were higher (*p* < 0.01) in the large intestine than in the forestomach and jejunum. For clarity and visualization purposes, the bacterial genera with a relative abundance of more than 0.5% are shown in a heatmap (Figure 4). The phylogenetic tree along the *X*‐axis in the upper part of Figure 4 revealed that the samples in the forestomach and large intestine clustered together, excluding the jejunum samples.

**Table 2:** Genus‐level microbial composition

Phylum	Genus	Rumen	Abomasum	Jejunum	Colon	Cecum
Bacteroidetes	*Prevotella*	20.44 ± 1.33^Aa^	19.68 ± 0.85^Ab^	2.14 ± 0.87^Bc^	0.20 ± 0.01^Cd^	0.29 ± 0.01^Cd^
	*Bacteroidales (Order)	18.82 ± 2.63^Aa^	10.72 ± 0.41^Bb^	2.92 ± 0.54^Dd^	7.89 ± 1.20^Cc^	7.73 ± 1.30^Cc^
Firmicutes	*Ruminococcaceae (Family)	4.81 ± 1.15^Bb^	4.89 ± 1.32^Bb^	4.96 ± 0.97^Bb^	24.83 ± 2.64^Aa^	23.86 ± 1.69^Aa^
	*Ruminococcus*	1.70 ± 0.26^Bc^	1.66 ± 0.17^Bc^	4.66 ± 1.13^Aa^	2.40 ± 0.69^Bb^	2.43 ± 0.81^Bb^
	*Clostridiales (Order)	5.02 ± 0.79^Cd^	6.29 ± 1.03^Cd^	23.20 ± 2.40^Aa^	11.47 ± 2.38^Bc^	13.06 ± 1.58^Bb^
	*Clostridiaceae (Family)	0.20 ± 0.07^Cc^	0.23 ± 0.04^Cc^	1.31 ± 0.43^Aa^	0.51 ± 0.14^Bb^	1.33 ± 0.22^Aa^
	*Clostridium*	0.34 ± 0.35^Cc^	0.37 ± 0.28^Cc^	0.49 ± 0.62^Cc^	7.28 ± 0.35^Bb^	7.61 ± 0.46^Aa^
	*Lachnospiraceae (Family)	4.33 ± 0.39^Bc^	3.51 ± 0.52^Cd^	4.56 ± 0.37^Bc^	6.80 ± 0.29^Aa^	5.29 ± 0.59^Ab^
Proteobacteria	*Butyrivibrio*	1.47 ± 0.19^Cc^	1.81 ± 0.09^Bb^	3.07 ± 0.51^Aa^	0.27 ± 0.06^Dd^	0.25 ± 0.02^Dd^
Fibrobacteres	*Fibrobacter*	0.65 ± 0.04^Bb^	1.50 ± 0.06^Aa^	0.39 ± 0.04^Cc^	0.66 ± 0.05^Bb^	0.65 ± 0.04^Bb^

Note

Relative abundances of the most abundant genera (genera whose relative abundance indicated that they were among the top 10 genera). Values are expressed as the *M*s ± *SD*. Values within the same column with same superscripts were not significantly different from one another (*p* > 0.05); however, values with different lowercase letter superscripts were significantly different (*p* < 0.05), and values with different capital letter superscripts were extremely significantly different (*p* < 0.01). Taxa that could not be assigned to a genus but were present in all samples were displayed using the highest taxonomic level that they could be assigned to.

### 3.4 Similarity analysis of the bacteria at the genus level

**Figure 5:**
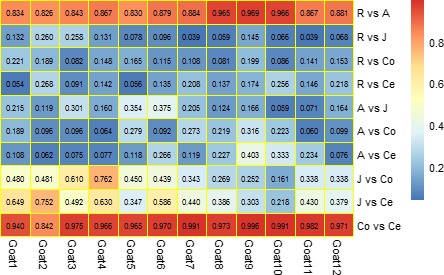
Similarity of the bacteria at the genus level. Pearson correlation analysis of the relative abundance of the bacterial community in the goat gastrointestinal tract. Only the taxa whose relative abundance was >0.1% of community are presented. *Note*. A correlation coefficient >0.5 indicates the existence of a correlation (*p* < 0.05), and that >0.7 indicates described a strong correlation (*p* < 0.01). R: rumen samples; A: abomasum samples; J: jejunum samples; Co: colon samples; and Ce: cecum samples

Statistical dissimilarities were observed across the GIT regions with respect to bacterial diversity (Figure 5). The results showed that the microbiota in the colon and cecum had the highest similarity, with Pearson correlation coefficients ranging from 0.842 to 0.996 (0.964 ± 0.041 on average); however, the microbiota in the rumen and colon had the lowest similarity, with Pearson correlation coefficients ranging from 0.081 to 0.221 (0.141 ± 0.047 on average). The Pearson correlation coefficient between the rumen and abomasum ranged from 0.826 to 0.969, with an average of 0.884 ± 0.053. Generally, the similarities between the microbial communities from adjacent GIT segments were higher than those between other regions.

**Figure 6:**
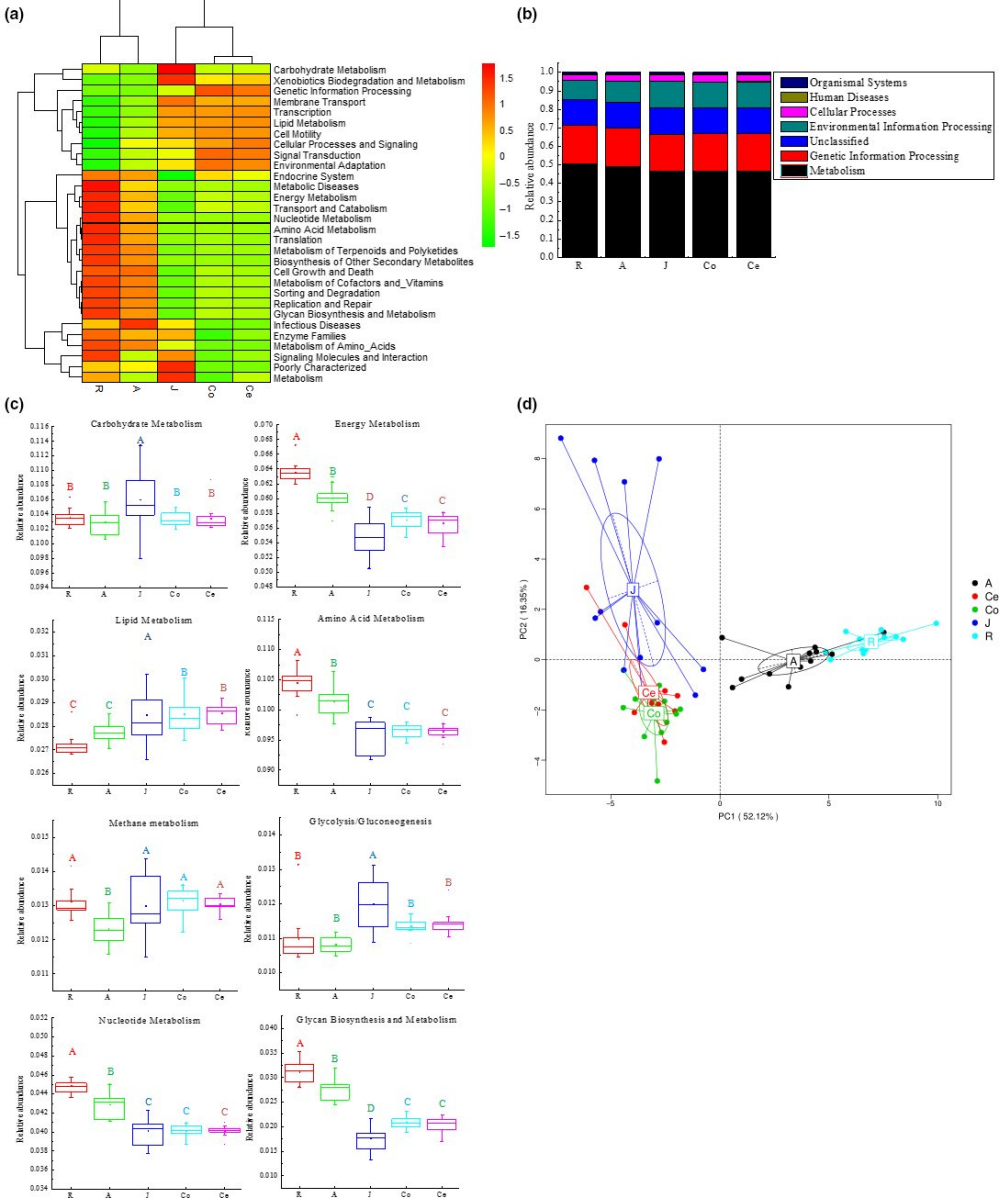
The majority of the gene sequences annotated to KEGG Level 3 orthologies, representing the predicted functional composition of the microbiota in goats (a) Heatmap of the functional gene distributions throughout the goat gastrointestinal tract (GIT) based on log transformed relative abundances. (b) Distribution of the dominant functional genes throughout the goat GIT. (c) Comparisons of the eight predominant metabolic pathways of the microbiota throughout the goat GIT. (d) Principal component analysis of microbial functional diversity across the goat GIT based on the relative abundances of the functional pathways. *Note*. R: rumen samples; A: abomasum samples; J: jejunum samples; Co: colon samples; Ce: cecum samples

The present study used PICRUSt to predict the molecular functions of each sample based on 16S rRNA data. PICRUSt is a bioinformatics tool that uses marker genes, in this case 16S rRNA, to predict the gene functional content of microorganisms. These predictions are precalculated for genes in databases including KEGG and COGs. The present study used the KEGG database and performed closed reference OTU picking using the sampled reads against Greengenes database. The potential function of the microbial communities across the goat GIT was predicted using PICRUSt. Forty‐one gene families such as amino acid metabolism, immune system diseases, cellular processes and signaling, circulatory system, and transport and catabolism, were found in all samples (KEGG Level 2 pathways). For clarity and visualization, the relative abundances of the top 30 gene families are shown in a heatmap (Figure 6a), which revealed that the samples in the forestomach clustered together, so did the large intestine samples, whereas the small intestine samples were separate from the others.

The majority of the genes predicted in all samples were involved in metabolism (Figure 6b) (KEGG Level 1 pathways), accounting for 50.29% ± 0.21%, 48.84 ± 0.24%, 46.47 ± 0.20%, 46.54 ± 0.15%, and 46.52 ± 0.11% of the total genes in the samples from the rumen, abomasum, jejunum, colon, and cecum samples, respectively. To better understand the differences among the gene families across the GIT, we compared the relative abundances of the eight predominant metabolic gene families in the whole GIT (Figure 6c). The results showed that these eight gene families were significantly different among the GIT regions (*p* < 0.002). Across the GIT regions, the forestomach had the highest (*p* < 0.01) abundance of genes involved in energy metabolism, amino acid metabolism, nucleotide metabolism, and glycan biosynthesis, while the small intestine possessed the lowest (*p* < 0.01) proportions of gene families involved in energy metabolism, amino acid metabolism, and glycan biosynthesis. In addition, the proportions of gene families involved in carbohydrate metabolism, lipid metabolism, methanogenesis, glycolysis, and gluconeogenesis were the highest (*p* < 0.01) in the small intestine, while those involved in carbohydrate metabolism, methanogenesis, glycolysis, and gluconeogenesis were the lowest (*p* < 0.01) in the abomasum.

The principal component analysis (PCA) on the relative abundance values of the KEGG pathways showed a clear distinction between the clustering of the forestomach and that of the intestinal tract samples (Figure. 6d). Furthermore, the results showed that the bacterial communities in the forestomach samples were clearly distinguished from those in other samples, as shown by PC1, which accounted for 52.12% of the total variation, and the bacterial communities in the large intestine samples were distinguished from those in the small intestine samples, as shown by PC2, which represented 16.35% of the total variation.

### 3.5 Correlation between the bacterial community and metabolic function

**Figure 7:**
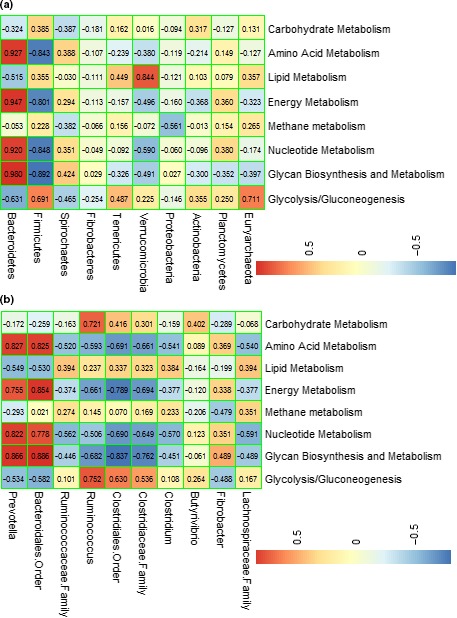
Correlation between the bacterial community and metabolic function. Pearson correlation matrix of the dominant bacteria at the (a) phylum and (b) genus level; the data presented represent the taxa with the top 10 relative abundances in the community. *Note*. An absolute value of the correlation coefficient >0.5 indicates the existence of correlation (*p* < 0.05), and that >0.7 indicates a strong correlation (*p* < 0.01). R: rumen samples; A: abomasum samples; J: jejunum samples; Co: colon samples; Ce: cecum samples

The main contributors to the abundant functional pathways were analyzed at the phylum and genus level (Figure 7). At the phylum level (Figure 7a), the relative abundance of Bacteroidetes was positively correlated with amino acid metabolism (*r* = 0.927, *p* < 0.01), nucleotide metabolism (*r* = 0.947, *p* < 0.01), energy metabolism (*r* = 0.920, *p* < 0.01), and glycan biosynthesis and metabolism (*r* = 0.980, *p* < 0.01). Conversely, the relative abundance of Firmicutes was negatively correlated with these metabolic functions (*r* = −0.843; *r* = −0.801; *r* = −0.848; *r* = −0.892; *p* < 0.01, respectively).

At the genus level (Figure 7b), *Prevotella* and Bacteroidales (order) (belonging to an Bacteroidetes‐OTU) were positively correlated with amino acid metabolism (*r* = 0.827, *p* < 0.01; *r* = 0.825, *p* < 0.01), nucleotide metabolism (*r* = 0.822, *p* < 0.01; *r* = 0.778, *p* < 0.01), energy metabolism (*r* = 0.755, *p* < 0.01; *r* = 0.854, *p* < 0.01), and glycan biosynthesis and metabolism (*r* = 0.866, *p* < 0.01; *r* = 0.886, *p* < 0.01). *Ruminococcus* (belonging to Firmicutes) was positively correlated with carbohydrate metabolism (*r* = 0.721, *p* < 0.01) and glycolysis/gluconeogenesis (*r* = 0.752, *p* < 0.01).

## 4 DISCUSSION

This study aimed to describe the compositions and the potential functions of the microbial communities across the GIT of goats using next‐generation sequencing technology. The results showed significant differences in the structures of the microbial communities among the GIT sections. For example, the most abundant phylum in the samples of the forestomach was Bacteroidetes, whereas that of the small and large intestine was Firmicutes. Additionally, the genus *Prevotella*, which was the main genus under the phylum Bacteroidetes, reached up to 20.44% and 19.48% of the total abundance in the rumen and abomasal samples, respectively (Table 2). The predominant genera in the small and large intestine microbiota were unclassified Clostridiales and unclassified Ruminococcaceae, respectively (Table 2), which belong to phylum Firmicutes. This finding agreed with those of previous studies (Frey et al., 2010; Stevenson & Weimer, 2007), in which the relative abundance of *Prevotella* was thought to be related to the genetic variability in the different compartments of the GIT. The reason that unclassified Clostridiales and unclassified Ruminococcaceae were enriched in the intestine is not clear yet, but the dominance of the genus *Prevotella* in the forestomach of goats was not unexpected (Abderzak et al., 2012; Huo, Zhu, & Mao, 2014; Riyanti et al., 2015). Compared to the other regions of the GIT in ruminants, the rumen is the place where nutrient digestion and metabolism mostly occur. Previous results showed that Bacteroidetes possess a strong ability to degrade protein and polysaccharides (Huo et al., 2014; Pitta et al., 2016), and these results were confirmed by the present study (Figure 7a). The genus *Prevotella* was found not only to degrade nonstructural carbohydrates and protein (Belanche et al., 2012; Purushe et al., 2010; Thompson, Monteagudomera, Cadenas, Lampl, & Azcarateperil, 2015) but also to be involved in amino acid metabolism, nucleotide metabolism, energy metabolism, and glycan biosynthesis, as revealed in this study (Figure 7b) and in a previous study (Hook et al., 2011) as well. In the present study, the family Lachnospiraceae was found in all the five compartments of the goat GIT. Previous studies showed that all species of the family Lachnospiraceae are anaerobic and can only be found in human and mammalian gut microbiota (Huynh et al., 2008). Our results also showed that Lachnospiraceae possessed a significantly higher relative abundance in the large intestine samples than in the forestomach and jejunum samples (Table 2). A higher abundance of Lachnospiraceae in the large intestine may be required to maintain the intestinal health of animals, because previous studies found that the family Lachnospiraceae acts as an indicator of large intestine health and some members can protect against colon cancer by producing butyric acid (Meehan & Beiko, 2014; Surana & Kasper, 2017). In addition, significant differences in the diversity and richness of bacteria among the GIT regions (Table 1 and Figure 6) were revealed by Simpson and Shannon indices as well as the PCoA plot (PC1 [40.74%] vs. PC2 [2.52%]) in the present study. All of our experimental results mentioned above indicated that significant differences in microbial diversity existed among the GIT sections. Previous studies have shown that the bacterial composition of animal GITs is mainly affected by animal species, age, sex, genetics, environment, and the dietary composition (Gong et al., 2017; Jiao et al., 2016; Mao et al., 2015; Wang et al., 2016). In the present study, all of these factors were consistent for the 12 experimental goats; however, the relative abundances of the dominant phyla and genera varied considerably among the GIT compartments, which emphasized that the sampling site was the major determinant of the microbial composition and community structure along the GIT. This phenomenon has been noticed by researchers in past scientific research reports (De Oliveira et al., 2013).

The composition of the bacterial community in different GIT sections also showed similarities in addition to differences. Samples from adjacent GIT compartments had more similar microbial communities than those from other segments (Figure 5). The microbial flora in the cecum and colon had the highest degree of similarity (0.964 ± 0.041), followed by that in the rumen and abomasum (0.884 ± 0.053). This result was in agreement with the result obtained from cattle studies (De Oliveira et al., 2013; Mao et al., 2015). These results may suggest that the similarities in the living environments (pH values, the gut motility, and secretion) of bacterial communities in adjacent compartments of the GIT explain the similarities in the microbiota in these regions (Turnbaugh et al., 2009). Additionally, because abomasal chyme comes from the rumen, a large number of ruminal bacteria flow into the abomasum with digesta, causing the similarity between the bacterial communities in the rumen and abomasum. The jejunum has a variable living environment of the microbiota that inhabit it, and this variability includes the dynamics of duodenal chyme and the pH changes caused by acidic chyme from the abomasum, which in turn leads to a lower level of similarity between the microbiota in the jejunum and other sections of the GIT. The colon and cecum, which are the two segments of the large intestine, are relatively closed, and the living environments of the bacteria are comparatively stable in these segments, allowing the microbiota of the colon and cecum to have the highest degree of similarity.

The microbiota in animal GITs has important biological functions, but the understanding of these aspects in goats is still limited. The present study analyzed the putative function of the bacterial community in the GIT of goats using PICRUSt. However, it should be emphasized that PICRUSt predictions are based on known functions of genes. Due to the limited number of studies on the functional genes of the bacterial community in goats, the predicted functions of the bacterial community in this study may be over‐ or underestimated. Based on the functions predicted by PICRUSt, at KEGG Level 3, many pathways related to metabolism were detected (Figure 6). The results showed that the most prevalent function could be categorized as metabolism (Figure 6b), agreeing with the results from previous studies (Lu et al., 2014; Ridaura et al., 2013). This finding can be explained by the fact that carbohydrates, proteins, and amino acids are essential ingredients for microbial growth (Erickson et al., 2012; Lamendella, Domingo, Ghosh, Martinson, & Oerther, 2011).

The present study showed that the metabolic functions of the bacteria in the goat GIT, such as carbohydrate metabolism, amino acid metabolism, and energy metabolism, were highly represented, which was consistent with the results from previous studies (Wetzels et al., 2015). The findings of the present study revealed significant differences (*p* < 0.002) in bacterial function among the GIT regions of goats (Figure 6c). For example, genes related to amino acid metabolism were more abundant in the rumen than in the small and large intestine. The rumen bacteria may possibly derive energy from amino acid fermentation (Malmuthuge et al., 2012), which implies that the bacteria in the rumen may be more necessary for amino acid degradation than that in other sections. Previous studies have also shown that the functional features of rumen bacteria are associated with the high expression of genes involved in nutrient metabolism, including amino acid metabolism (Mann, Wetzels, Wagner, Zebeli, & Schmitz‐Esser, 2018; Wang, Elekwachi, et al., 2017; Wang, Liu, Yin, Zhu, & Mao, 2017). Moreover, the results from the PCA (Figure 6d) revealed significant differences (PC1 [52.12%] vs. PC2 [16.35%]) in metabolic functions across the goat GIT, which indicated that the bacterial community in the GIT was the determinant of metabolic function. The microbiota in the small and large intestine have not been studied as frequently as that in the rumen. The present study showed that the relative abundances of *Ruminococcus* and *Butyrivibrio* in the jejunum samples were significantly higher (*p* < 0.001) than those in the rumen samples (Table 2). Previous studies have shown that *Ruminococcus* and *Butyrivibrio* are important in carbohydrate metabolism in the GIT (Stevenson & Weimer, 2007), and those results were verified in the present study (Figure 7). These results suggested that the jejunum may also participate in carbohydrate metabolism, and previous studies also have shown that the intestines of ruminants can compensate for the carbohydrate metabolism that mainly occurs in the forestomach (Wang, Elekwachi, et al., 2017; Wang, Liu, Yin, Zhu, & Mao, 2017; Zoetendal et al., 2012). Therefore, enhanced *Ruminococcus* and *Butyrivibrio* in the small intestine may increase the bioavailability of carbohydrate for the host.

## 5 CONCLUSION

In general, this research revealed the composition and diversity, and partially revealed the potential functions of the microbial communities across the goat GIT. The microbes differed greatly by GIT region and that there were similarities between the adjacent GIT segments. These findings can be potentially used to modulate gastrointestinal microbiota and therefore improve the health and nutrient utilization of goats.

## CONFLICT OF INTERESTS

The authors declare no conflict of interest.

## AUTHORS CONTRIBUTION

L.Z., L.J., and B.X. designed the experiments; L.Z., L.J., Z.W., and Q.P. performed the experiments; B.X., Z.W., and Q.P. contributed reagents/materials/analysis tools, all authors analyzed the data; L.Z. and L.J. wrote the manuscript. All authors read the final manuscript.

## ETHICS STATEMENT

The experimental protocol used in the present study was approved by the Animal Policy and Welfare Committee of the Agricultural Research Organization of Sichuan Province, China and was in accordance with the guidelines of the Animal Care and Ethical Committee of the Sichuan Agricultural University.

## Data Availability

All sequence data in the present study were deposited in the sequence read archive (SRA) of the NCBI database under the number SRP185613.

## References

[mbo3820-bib-0001] Abderzak, L. , Pierre, N. , Mathieu, S. , Morgavi, D. P. , Claudette, B. , & Cécile, M. (2012). Rumen microbial and fermentation characteristics are affected differently by bacterial probiotic supplementation during induced lactic and subacute acidosis in sheep. BMC Microbiology, 12, 142 10.1186/1471-2180-12-142 22812531 PMC3438074

[mbo3820-bib-0002] Bauer, P. V. , Duca, F. A. , Waise, T. Z. , Rasmussen, B. A. , Abraham, M. A. , Dranse, H. J. , … Lam, T. K. (2018). Metformin alters upper small intestinal microbiota that impact a glucose‐SGLT1‐sensing glucoregulatory pathway. Cell Metabolism, 27, 101–117. 10.1016/j.cmet.2017.09.019 29056513

[mbo3820-bib-0003] Belanche, A. , Doreau, M. , Edwards, J. E. , Moorby, J. M. , Pinloche, E. , & Newbold, C. J. (2012). Shifts in the rumen microbiota due to the type of carbohydrate and level of protein ingested by dairy cattle are associated with changes in rumen fermentation. Journal of Nutrition, 142, 1684–1692. 10.3945/jn.112.159574 22833657

[mbo3820-bib-0004] Caporaso, J. G. , Kuczynski, J. , & Stombaugh, J. (2010). QIIME allows analysis of high‐throughput community sequencing data. Nature Methods, 7, 335–336. 10.1038/nmeth.f.303 20383131 PMC3156573

[mbo3820-bib-0005] Caporaso, J. G. , Lauber, C. L. , Walters, W. A. , Berglyons, D. , Huntley, J. , Fierer, N. , … Gormley, N. (2012). Ultra‐high‐throughput microbial community analysis on the illumina HiSeq and MiSeq platforms. The ISME Journal, 6, 1621–1624. 10.1038/ismej.2012.8 22402401 PMC3400413

[mbo3820-bib-0006] Caporaso, J. G. , Lauber, C. L. , Walters, W. A. , Berg‐Lyons, D. , Lozupone, C. A. , Turnbaugh, P. J. , … Knight, R. (2011). Global patterns of 16s rRNA diversity at a depth of millions of sequences per sample. Proceedings of the National Academy of Sciences of the United States of America, 108(Suppl 1), 4516–4522. 10.1073/pnas.1000080107 20534432 PMC3063599

[mbo3820-bib-0007] Cervantesbarragan, L. , Chai, J. N. , Tianero, M. D. , Diluccia, B. , Ahern, P. P. , Merriman, J. , … Cella, M. (2017). Lactobacillus reuteri induces gut intraepithelial CD4^+^ CD8αα^+^ t cells. Science, 357, 806 10.1126/science.aah5825 28775213 PMC5687812

[mbo3820-bib-0008] Cole, J. R. , Wang, Q. , Cardenas, E. , Fish, J. , Chai, B. , Farris, R. J. , … Tiedje, J. M. (2009). The ribosomal database project: Improved alignments and new tools for rRNA analysis. Nucleic Acids Research, 37, D141–D145. 10.1093/nar/gkn879 19004872 PMC2686447

[mbo3820-bib-0009] De Oliveira, M. N. , Jewell, K. A. , Freitas, F. S. , Benjamin, L. A. , Tótola, M. R. , Borges, A. C. , … Suen, G. (2013). Characterizing the microbiota across the gastrointestinal tract of a Brazilian Nelore steer. Veterinary Microbiology, 164, 307–314. 10.1016/j.vetmic.2013.02.013 23490556

[mbo3820-bib-0010] Dodd, D. , Spitzer, M. H. , Van, W. T. , Merrill, B. D. , Hryckowian, A. J. , Higginbottom, S. K. , … Sonnenburg, J. L. (2017). A gut bacterial pathway metabolizes aromatic amino acids into nine circulating metabolites. Nature, 3, e00438 10.1038/nature24661 PMC5850949 29168502

[mbo3820-bib-0011] Dougal, K. , Harris, P. A. , Edwards, A. , Pachebat, J. A. , Blackmore, T. M. , Worgan, H. J. , & Newbold, C. J. (2012). A comparison of the microbiome and the metabolome of different regions of the equine hindgut. FEMS Microbiology Ecology, 82, 642–652. 10.1111/j.1574-6941.2012.01441.x 22757649

[mbo3820-bib-0012] Edgar, R. C. (2010). Search and clustering orders of magnitude faster than blast. Bioinformatics, 26, 2460 10.1093/bioinformatics/btq461 20709691

[mbo3820-bib-0013] Edgar, R. C. , Haas, B. J. , Clemente, J. C. , Quince, C. , & Knight, R. (2011). Uchime improves sensitivity and speed of chimera detection. Bioinformatics, 27, 2194 10.1093/bioinformatics/btr381 21700674 PMC3150044

[mbo3820-bib-0015] Erickson, A. R. , Cantarel, B. L. , Lamendella, R. , Darzi, Y. , Mongodin, E. F. , Pan, C. , … Raes, J. (2012). Integrated metagenomics/metaproteomics reveals human host‐microbiota signatures of Crohn's disease. PLoS ONE, 7, e49138 10.1371/journal.pone.0049138 23209564 PMC3509130

[mbo3820-bib-0016] Frey, J. C. , Pell, A. N. , Berthiaume, R. , Lapierre, H. , Lee, S. , Ha, J. K. , … Angert, E. R. (2010). Comparative studies of microbial populations in the rumen, duodenum, ileum and faeces of lactating dairy cows. Journal of Applied Microbiology, 108, 1982–1993. 10.1111/j.1365-2672.2009.04602.x 19863686

[mbo3820-bib-0017] Fu, L. , Jiang, B. , Liu, J. , Zhao, X. , Liu, Q. , & Hu, X. (2016). Genome sequence analysis of a flocculant‐producing bacterium, *Paenibacillus shenyangensis* . Biotechnology Letters, 38(3), 1–7. 10.1007/s10529-015-1990-2 26573635

[mbo3820-bib-0018] Gong, Y. , Guo, H. , Zhang, Z. , Zhou, H. , Zhao, R. , & He, B. (2017). Heat stress reduces sperm motility via activation of glycogen synthase kinase‐3α and inhibition of mitochondrial protein import. Frontiers in Physiology, 8, 718 10.3389/fphys.2017.00718 29018353 PMC5615227

[mbo3820-bib-0019] Gu, S. , Chen, D. , Zhang, J. N. , Lv, X. , Wang, K. , Duan, L. P. , … Wu, X. L. (2013). Bacterial community mapping of the mouse gastrointestinal tract. PLoS ONE, 8, e74957 10.1371/journal.pone.0074957 24116019 PMC3792069

[mbo3820-bib-0020] Guo, W. , Li, Y. , Wang, L. , Wang, J. , Xu, Q. , Yan, T. , & Xue, B. (2015). Evaluation of composition and individual variability of rumen microbiota in yaks by 16s rRNA high‐throughput sequencing technology. Anaerobe, 34, 74–79. 10.1016/j.anaerobe.2015.04.010 25911445

[mbo3820-bib-0021] Hook, S. E. , Steele, M. A. , Northwood, K. S. , Dijkstra, J. , France, J. , Wright, A. D. , & McBride, B. W. (2011). Impact of subacute ruminal acidosis (SARA) adaptation and recovery on the density and diversity of bacteria in the rumen of dairy cows. FEMS Microbiology Ecology, 78, 275–284. 10.1111/j.1574-6941.2011.01154.x 21692816

[mbo3820-bib-0022] Huo, W. , Zhu, W. , & Mao, S. (2014). Impact of subacute ruminal acidosis on the diversity of liquid and solid‐associated bacteria in the rumen of goats. World Journal of Microbiology & Biotechnology, 30, 669–680. 10.1007/s11274-013-1489-8 24068532

[mbo3820-bib-0023] Huse, S. M. , Welch, D. M. , Morrison, H. G. , & Sogin, M. L. (2010). Ironing out the wrinkles in the rare biosphere through improved OTU clustering. Environmental Microbiology, 12, 1889–1898. 10.1111/j.1462-2920.2010.02193.x 20236171 PMC2909393

[mbo3820-bib-0024] Huynh, L. Y. , Ert, M. N. V. , Hadfield, T. , Probert, W. S. , Bellaire, B. H. , & Dobson, M. , … Keim, P. (2008). Multiple locus variable number tandem repeat (VNTR) analysis (MLVA) of *Brucella* spp. identifies species‐specific markers and insights into phylogenetic relationships In Georgiev V. S., Western K. A., & McGowan J. J. (Eds.), National institute of allergy and infectious diseases (pp. 47–54). Totowa, NJ: Humana Press 10.1007/978-1-59745-569-5_6

[mbo3820-bib-0025] Jiao, J. , Wu, J. , Zhou, C. , Tang, S. , Wang, M. , & Tan, Z. (2016). Composition of ileal bacterial community in grazing goats varies across non‐rumination, transition and rumination stages of life. Frontiers in Microbiology, 7, 1364 10.3389/fmicb.2016.01364 27656165 PMC5011132

[mbo3820-bib-0026] Kadoki, M. , Patil, A. , Thaiss, C. C. , Brooks, D. J. , Pandey, S. , Deep, D. , … Mikkelsen, T. S. (2017). Organism‐level analysis of vaccination reveals networks of protection across tissues. Cell, 171, 398 10.1016/j.cell.2017.08.024 28942919 PMC7895295

[mbo3820-bib-0027] Koppel, N. , Maini, R. V. , & Balskus, E. P. (2017). Chemical transformation of xenobiotics by the human gut microbiota. Science, 356, 1246–1257. 10.1126/science.aag2770 PMC5534341 28642381

[mbo3820-bib-0028] Lamendella, R. , Domingo, J. W. S. , Ghosh, S. , Martinson, J. , & Oerther, D. B. (2011). Comparative fecal metagenomics unveils unique functional capacity of the swine gut. BMC Microbiology, 11, 103 10.1186/1471-2180-11-103 21575148 PMC3123192

[mbo3820-bib-0029] Langille, M. G. , Zaneveld, J. , Caporaso, J. G. , Mcdonald, D. , Knights, D. , Reyes, J. A. , … Beiko, R. G. (2013). Predictive functional profiling of microbial communities using 16s rRNA marker gene sequences. Nature Biotechnology, 31, 814 10.1038/nbt.2676 PMC3819121 23975157

[mbo3820-bib-0030] Ley, R. E. , Hamady, M. , Lozupone, C. , Turnbaugh, P. J. , Ramey, R. R. , Bircher, J. S. , … Gordon, J. L. (2008). Bircher, evolution of mammals and their gut microbes. Science, 320, 1647–1651. 10.1126/science.1155725 18497261 PMC2649005

[mbo3820-bib-0031] Liu, X. , Fan, H. , Ding, X. , Hong, Z. , Nei, Y. , Liu, Z. , … Guo, H. (2014). Analysis of the gut microbiota by high‐throughput sequencing of the v5–v6 regions of the 16s rRNA gene in donkey. Current Microbiology, 68, 657–662. 10.1007/s00284-014-0528-5 24452427

[mbo3820-bib-0032] Lozupone, C. , Lladser, M. E. , Knights, D. , Stombaugh, J. , & Knight, R. (2011). Unifrac: An effective distance metric for microbial community comparison. The ISME Journal, 5, 169–172. 10.1038/ismej.2010.133 20827291 PMC3105689

[mbo3820-bib-0033] Lu, K. , Abo, R. P. , Schlieper, K. A. , Graffam, M. E. , Levine, S. , Wishnok, J. S. , … Fox, J. G. (2014). Arsenic exposure perturbs the gut microbiome and its metabolic profile in mice: An integrated metagenomics and metabolomics analysis. Environmental Health Perspectives, 122, 284 10.1289/ehp.1307429 24413286 PMC3948040

[mbo3820-bib-0034] Malmuthuge, N. , Li, M. , Chen, Y. , Fries, P. , Griebel, P. J. , Baurhoo, B. , … Guan, L. L. (2012). Distinct commensal bacteria associated with ingesta and mucosal epithelium in the gastrointestinal tracts of calves and chickens. FEMS Microbiology Ecology, 79, 337–347. 10.1111/j.1574-6941.2011.01220.x 22092450

[mbo3820-bib-0035] Mann, E. , Wetzels, S. U. , Wagner, M. , Zebeli, Q. , & Schmitz‐Esser, S. (2018). Metatranscriptome sequencing reveals insights into the gene expression and functional potential of rumen wall bacteria. Frontiers in Microbiology, 9, 43 10.3389/fmicb.2018.00043 29410661 PMC5787071

[mbo3820-bib-0036] Mao, S. , Zhang, M. , Liu, J. , & Zhu, W. (2015). Characterising the bacterial microbiota across the gastrointestinal tracts of dairy cattle: Membership and potential function. Scientific Reports, 5, 16116 10.1038/srep16116 26527325 PMC4630781

[mbo3820-bib-0037] Meehan, C. J. , & Beiko, R. G. (2014). A phylogenomic view of ecological specialization in the lachnospiraceae, a family of digestive tract‐associated bacteria. Genome Biology and Evolution, 6, 703–713. 10.1093/gbe/evu050 24625961 PMC3971600

[mbo3820-bib-0038] Pitta, D. W. , Pinchak, W. E. , Indugu, N. , Vecchiarelli, B. , Sinha, R. , & Fulford, J. D. (2016). Metagenomic analysis of the rumen microbiome of steers with wheat‐induced frothy bloat. Frontiers in Microbiology, 7, 689 10.3389/fmicb.2016.00689 27242715 PMC4863135

[mbo3820-bib-0039] Purushe, J. , Fouts, D. E. , Morrison, M. , White, B. A. , Mackie, R. I. , Coutinho, P. M. , … North American Consortium for Rumen Bacteria . (2010). Comparative genome analysis of prevotella ruminicola and *Prevotella bryantii*: Insights into their environmental niche. Microbial Ecology, 60, 721–729. 10.3389/fmicb.2016.00689 20585943

[mbo3820-bib-0040] Quast, C. , Pruesse, E. , Yilmaz, P. , Gerken, J. , Schweer, T. , Yarza, P. , … Glöckner, F. O. (2013). The silva ribosomal rna gene database project: Improved data processing and web‐based tools. Nucleic Acids Research, 41, 590–596. 10.1093/nar/gks1219 PMC3531112 23193283

[mbo3820-bib-0041] Ramírez‐Restrepo, C. A. , Tan, C. , O'Neill, C. J. , López‐Villalobos, N. , Padmanabha, J. , Wang, J. , & McSweeney, C. S. (2016). Methane production, fermentation characteristics, and microbial profiles in the rumen of tropical cattle fed tea seed saponin supplementation. Animal Feed Science & Technology, 216, 58–67. 10.1016/j.anifeedsci.2016.03.005

[mbo3820-bib-0042] Ridaura, V. K. , Faith, J. J. , Rey, F. E. , Cheng, J. , Duncan, A. E. , Kau, A. L. , … Muehlbauer, M. J. (2013). Gut microbiota from twins discordant for obesity modulate metabolism in mice. Science, 341, 1079‐U49 10.1126/science.1241214 PMC3829625 24009397

[mbo3820-bib-0043] Riyanti, L. , Suryahadi , & Evvyernie, D. (2015). In vitro fermentation characteristics and rumen microbial population of diet supplemented with *Saccharomyces cerevisiae* and rumen microbe probiotics. Media Peternakan, 39(1), 40–45. 10.5398/medpet.2016.39.1.40

[mbo3820-bib-0044] Schloss, P. D. , Westcott, S. L. , Ryabin, T. , Hall, J. R. , Hartmann, M. , Hollister, E. B. , … Sahl, J. W. (2009). Introducing mothur: Open‐source, platform‐independent, community‐supported software for describing and comparing microbial communities. Applied & Environmental Microbiology, 75, 7537 10.1128/AEM.01541-09 19801464 PMC2786419

[mbo3820-bib-0045] Stevenson, D. M. , & Weimer, P. J. (2007). Dominance of prevotella, and low abundance of classical ruminal bacterial species in the bovine rumen revealed by relative quantification real‐time PCR. Applied Microbiology & Biotechnology, 75, 165–174. 10.1007/s00253-006-0802-y 17235560

[mbo3820-bib-0046] Surana, N. K. , & Kasper, D. L. (2017). Moving beyond microbiome‐wide associations to causal microbe identification. Nature, 552, 244–247. 10.1038/nature25019 29211710 PMC5730484

[mbo3820-bib-0047] Thompson, A. L. , Monteagudomera, A. , Cadenas, M. B. , Lampl, M. L. , & Azcarateperil, M. A. (2015). Milk‐ and solid‐feeding practices and daycare attendance are associated with differences in bacterial diversity, predominant communities, and metabolic and immune function of the infant gut microbiome. Frontiers in Cellular and Infection Microbiology, 5, 3 10.3389/fcimb.2015.00003 25705611 PMC4318912

[mbo3820-bib-0048] Turnbaugh, P. J. , Ridaura, V. K. , Faith, J. J. , Rey, F. E. , Knight, R. , & Gordon, J. I. (2009). The effect of diet on the human gut microbiome: A metagenomic analysis in humanized gnotobiotic mice. Science Translational Medicine, 1, 6ra14 10.1126/scitranslmed.3000322 PMC2894525 20368178

[mbo3820-bib-0049] Wang, Z. , Elekwachi, C. , Jiao, J. , Wang, M. , Tang, S. , Zhou, C. , … Forster, R. J. (2017). Changes in metabolically active bacterial community during rumen development, and their alteration by rhubarb root powder revealed by 16s rRNA amplicon sequencing. Frontiers in Microbiology, 8, 159 10.3389/fmicb.2017.00159 28223972 PMC5293741

[mbo3820-bib-0050] Wang, Y. , Liu, J. , Yin, Y. , Zhu, W. , & Mao, S. (2017). Rumen microbial and fermentation characteristics are affected differently by acarbose addition during two nutritional types of simulated severe subacute ruminal acidosis in vitro. Anaerobe, 47, 39–46. 10.1016/j.anaerobe.2017.04.003 28392309

[mbo3820-bib-0051] Wang, L. , Xu, Q. , Kong, F. , Yang, Y. , Wu, D. , Mishra, S. , & Li, Y. (2016). Exploring the goat rumen microbiome from seven days to two years. PLoS ONE, 11, e0154354 10.1371/journal.pone.0154354 27135948 PMC4852915

[mbo3820-bib-0052] Wetzels, S. U. , Mann, E. , Metzler‐Zebeli, B. U. , Wagner, M. , Klevenhusen, F. , Zebeli, Q. , & Schmitz‐Esser, S. (2015). Pyrosequencing reveals shifts in the bacterial epimural community relative to dietary concentrate amount in goats. Journal of Dairy Science, 98, 5572–5587. 10.3168/jds.2014-9166 26051320

[mbo3820-bib-0053] Zhao, L. L. , Wang, G. , Siegel, P. , He, C. , Wang, H. Z. , Zhao, W. J. , … Sun, Z. (2013). Quantitative genetic background of the host influences gut microbiomes in chickens. Scientific Reports, 3, 1163 10.1038/srep01163 23362462 PMC3557447

[mbo3820-bib-0054] Zoetendal, E. G. , Raes, J. , Bogert, B. V. D. , Arumugam, M. , Booijink, C. C. , Troost, F. J. , … Kleerebezem, M. (2012). The human small intestinal microbiota is driven by rapid uptake and conversion of simple carbohydrates. The ISME Journal, 6, 1415–1426. 10.1038/ismej.2011.212 22258098 PMC3379644

